# Supplementary material for: Sequential action of a tRNA base editor in conversion of cytidine to pseudouridine
Source: Nat Commun. 2022 Oct 11;13:5994. doi: 10.1038/s41467-022-33714-x (PMC9553926; doi:10.1038/s41467-022-33714-x)
Supplement: Supplementary file 1 — Supplementary Information [file 41467_2022_33714_MOESM1_ESM.pdf]

Supplementary Information

Sequential action of a tRNA base editor in conversion of cytidine to pseudouridine

Satoshi Kimura[1,2,3,*], Veerasak Srisuknimit[1,2,3], Kacie McCarty[2,4], Peter C. Dedon[5,6], Philip J. Kranzusch[2,4] and Matthew K. Waldor[1,2,3,*]

[1]Division of Infectious Diseases, Brigham and Women's Hospital, Boston, Massachusetts, USA
[2]Department of Microbiology, Harvard Medical School, Boston, Massachusetts, USA
[3]Howard Hughes Medical Institute, Boston, Massachusetts, USA
[4]Department of Cancer Immunology and Virology, Dana-Farber Cancer Institute, Boston, Massachusetts, USA
[5]Department of Biological Engineering, Massachusetts Institution of Technology, Cambridge, Massachusetts, USA
[6]Singapore-MIT Alliance for Research and Technology Antimicrobial Resistance Interdisciplinary Research Group

For correspondence: s.kimura.res@gmail.com or mwaldor@research.bwh.harvard.edu

ADAR family (Adenosine deaminase/Editase domain)

Substrate	Protein and organism	Amino acid sequence							
mRNA	ADAR2_HUMAN	392-	D C H A E	-52-	S P C G D	-100-	M S C S D K	-559
	ADAR_DROME	370-	D S H A E	-53-	A P C G D	-58-	M S C S D K	-496
	ADAR1_HUMAN	908-	D C H A E	-51-	A P C G D	-65-	M S C S D K	-1039
	ADAR2_CAEEL	189-	D C H A E	-48-	A P C G V	-56-	M S C S D K	-308
tRNA	ADAT1_human	85-	D S H A E	-50-	T P C G D	-152-	M S C S D K	-302
	ADAT1_DROME	76-	D S H A E	-44-	T P C G D	-59-	M S C S D K	-194

ADAT, CDA, and APOBEC/AID family (Cytidine and deoxycytidylate deaminase domain)

Substrate	Protein and organism	Amino acid sequence				
dCMP	DCTD_YEAST	231-	C L H A E	-22-	C P C L T C S V	-265
	DCTD_HUMAN	82-	V C H A E	-21-	F P C N E C A K	-115
tRNA	TADA_ECOLI	55-	T A H A E	-25-	E P C V M C A G	-92
	TAD2_YEAST	52-	V A H A E	-29-	E P C I M C A S	-93
	ADAT2_HUMAN	68-	T R H A E	-31-	E P C I M C A A	-112
Cytidine	CDD_HUMAN	63-	G I C A E	-29-	S P C G A C R Q	-104
	CDD_YEAST	59-	C I C A E	-30-	S P C G V C R Q	-101
Blasticidin-S	BSD_COCIM	55-	G P C A E	-29-	S P C G R C R Q	-96
mRNA, DNA	APOBEC1_HUMAN	59-	T N H V E	-27-	S P C W E C S Q	-98
	APOBEC2_HUMAN	96-	A A H A E	-25-	S P C A A C A D	-133
	APOBEC3C_HUMAN	64-	H C H A E	-26-	S P C P D C A G	-102
	AICDA_HUMAN	54-	G C H V E	-26-	S P C Y D C A R	-92

TrcP-CDA

Organism	Amino acid sequence						
Shewanella putrefaciens	288-	G E C A A	-16-	I A E F W W G	-14-	P A C R R K C H P	-338
Pseudoalteromonas tunicata	278-	G E C A A	-16-	M A E F W W G	-14-	P A C L S K C Q P	-328
Vibrio cholerae	291-	G E C A A	-16-	L A E F W W G	-14-	P A C Q S K C Q P	-341
Flavobacterium cauense	287-	G E C A A	-16-	M A E F W W G	-14-	P A C R G K C E P	-337
Bacteroides fragilis	260-	G E C A A	-16-	M A E F W W G	-14-	P S C K G K C G P	-310
Myxococcus xanthus	277-	A D C A G	-16-	L A E F W W G	-14-	P A C K D K C G P	-327
Microcystis aeruginosa	239-	G E C C A	-16-	M A E F W W G	-12-	G A C Q E R C Q P	-287
Synechocystis	211-	G E C C A	-16-	I A E F W W G	-12-	G A C A E R C Q P	-259

Supplementary Fig. 1 Sequence motifs in deaminases.

Conserved histidine/cysteine clusters for coordination of zinc are highlighted in yellow. The catalytic glutamate residue is highlighted in light blue. The substrate molecules for the ADAR, ADAT, CDA, and APOBEC/AID families are shown on the left. The amino acids that are identical between all sequences are shown in bold. The numbers represent the first and last amino acid positions of the retrieved sequences and the length of the amino acids between conserved motifs.

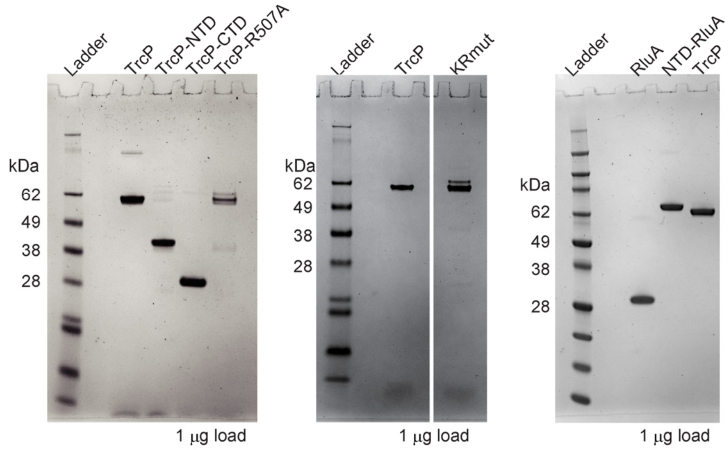

Supplementary Fig. 2 Purified recombinant proteins

Coomassie-stained gels of purified proteins; 1 µg of each protein is loaded. This experiment was performed once. Uncropped blots are provided in Source Data.

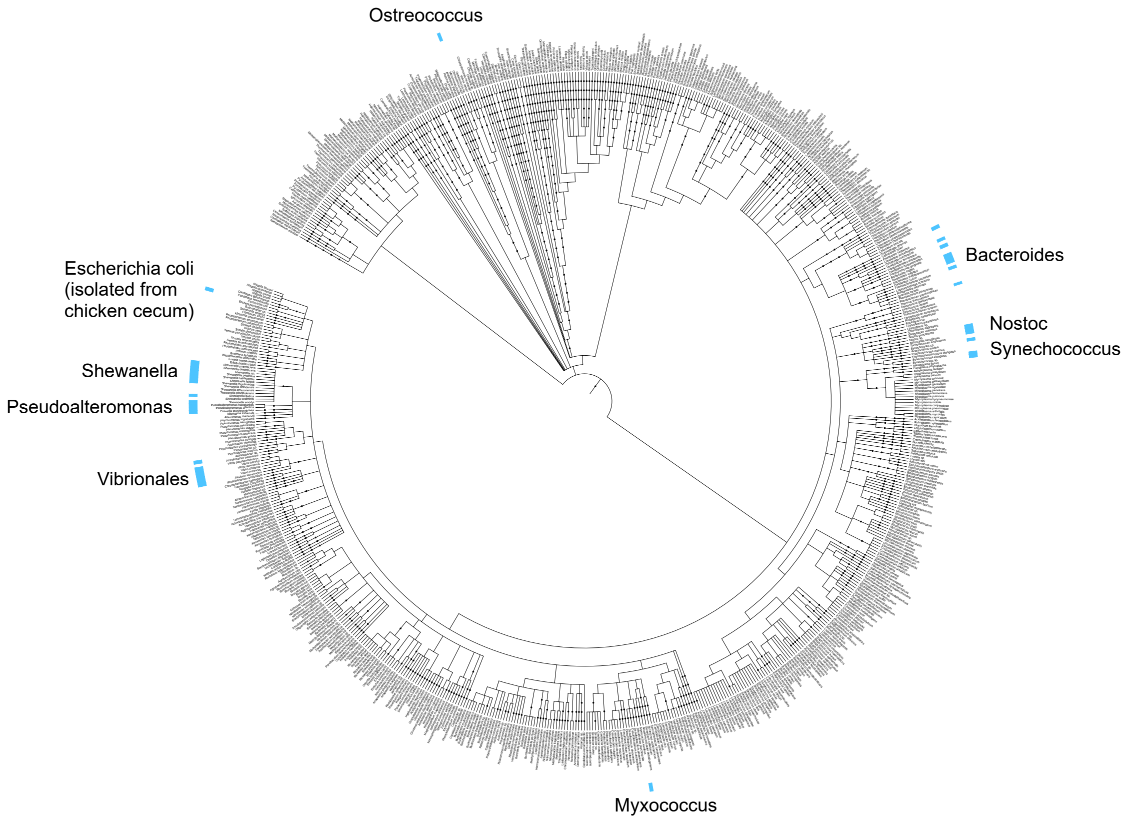

Supplementary Fig. 3 Phylogenetic tree with the distribution of TrcP homologs
The organisms bearing TrcP homologs are indicated with light blue bars

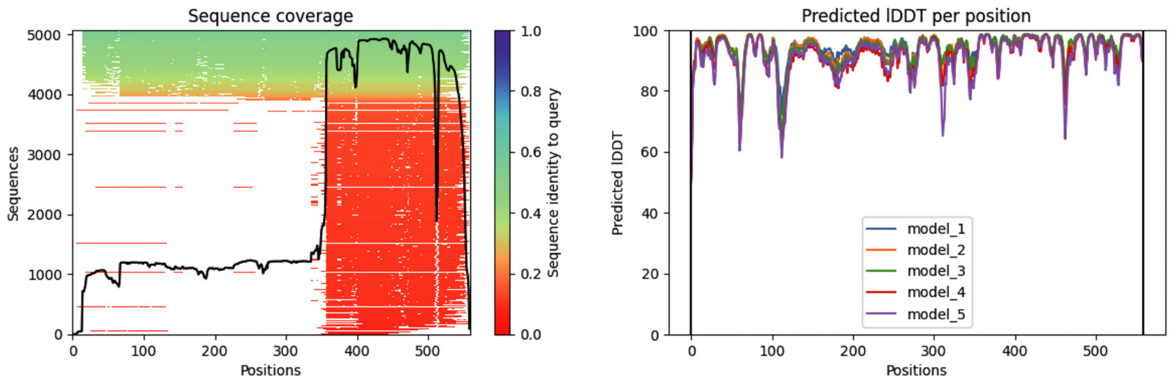

Supplementary Fig. 4 Quality scores of structural predictions by ColabFold

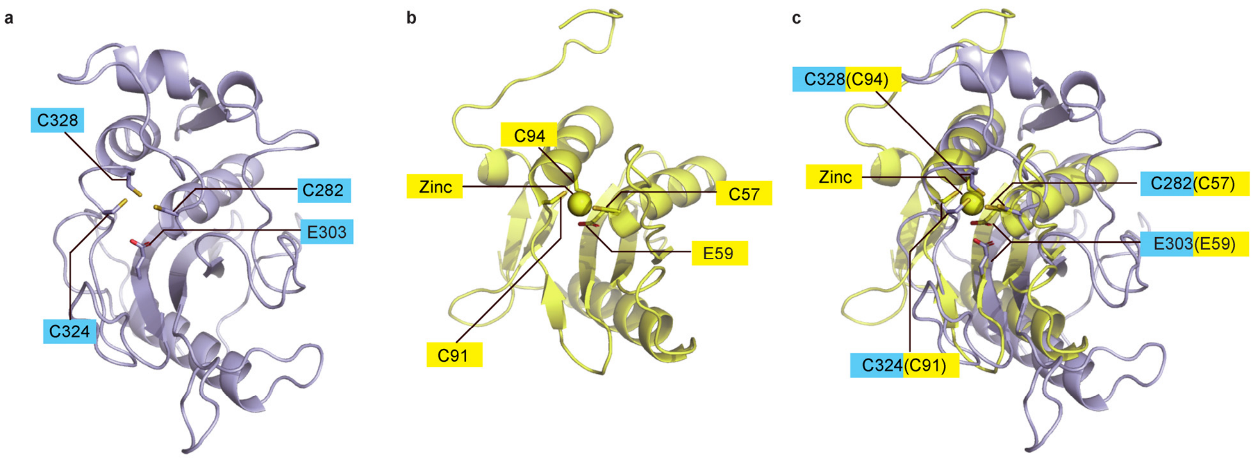

Supplementary Fig. 5 Comparison between the predicted structures of TrcP and Blasticidin-S deaminase (BSD)

a. Predicted structure of the TrcP-CDA domain. The conserved cysteine and glutamate residues are indicated.

b. Structure of Blasticidin-S deaminase (PDB 3oj6). The cysteine cluster and conserved glutamate residue in the catalytic site and zinc ion are shown.

c. A structural alignment of TrcP-CDA domain and BSD. The conserved cysteines, glutamate and zinc ion are shown.

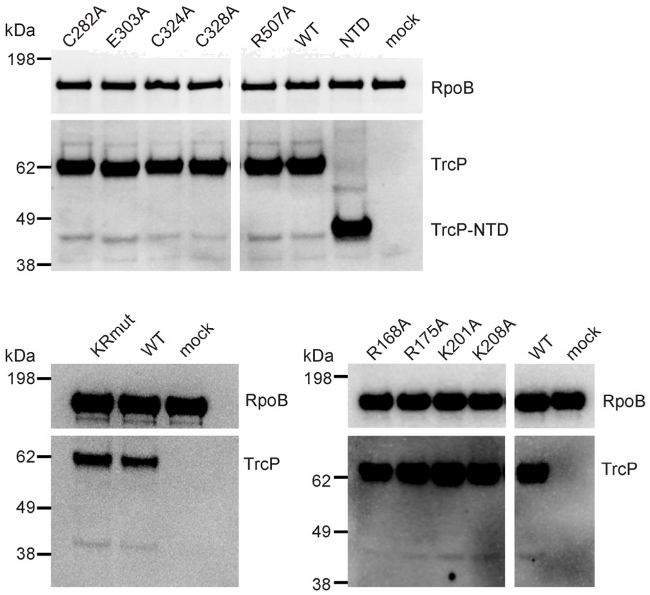

Supplementary Fig. 6 Expression levels of mutant TrcP proteins in strains used for *in vivo* complementation assays. Uncropped blots are provided in Source Data. This experiment was performed once.

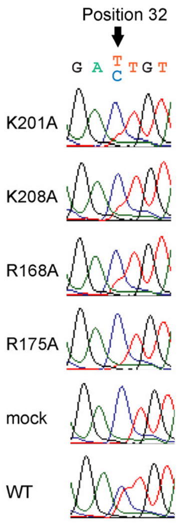

Supplementary Fig. 7 *In vivo* complementation assay with single mutants of the positively charged patch

Sanger sequencing results of tRNA-Tyr cDNA derived from RNA isolated from *trcP* knockout strains expressing TrcP (WT), mutant derivatives, and empty vector (mock).

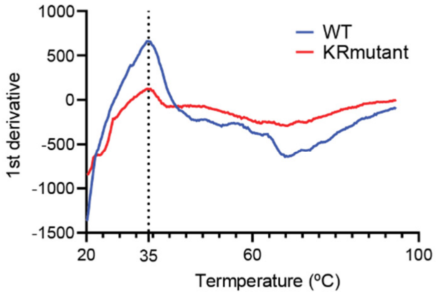

Supplementary Fig. 8 First derivative lines of melting curves of TrcP proteins

Sypro Orange dye was mixed with proteins, and the fluorescence signals were measured using the filter for FAM signals as the temperature was raised. The first derivative curves were calculated by Graphpad Prism (2nd order smoothing neighboring 8 points), and the average lines of three reactions are depicted. The melting temperatures represented by the peaks of the lines are very similar (35°C) between WT and KRmutant. Source data are provided as a Source Data File.

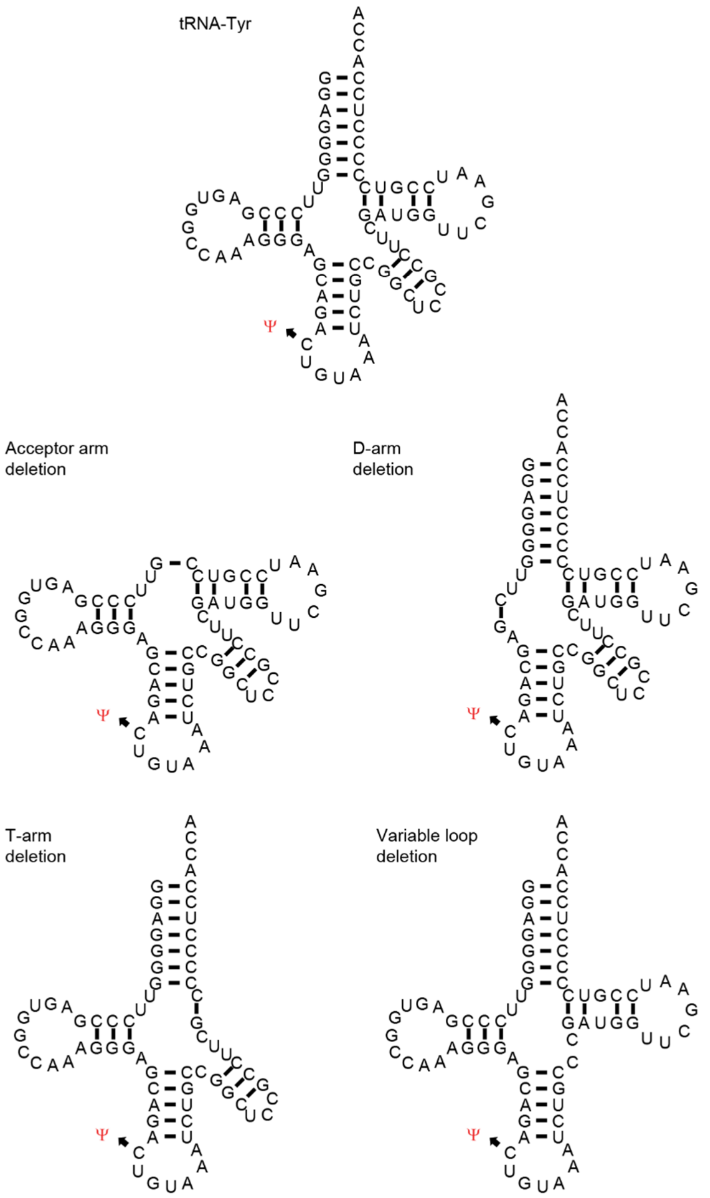

Supplementary Fig. 9 Secondary structures of tRNA-Tyr mutants tested in Figure. 4D

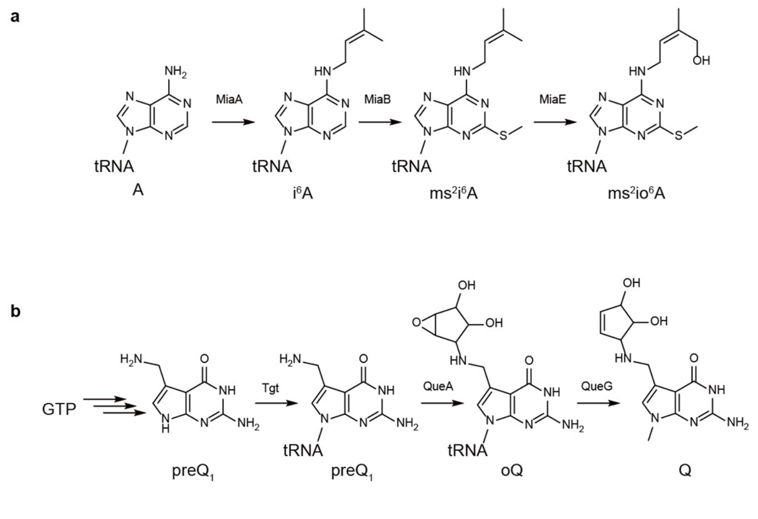

Supplementary Fig. 10 Biosynthesis pathways of ms²io⁶A (a) and Q (b)

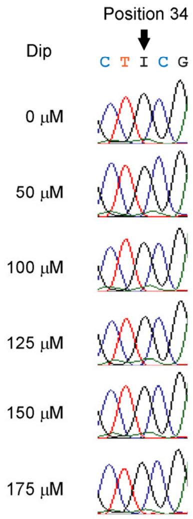

Supplementary Fig. 11 A-to-I editing frequencies upon iron depletion

A-to-I editing occurs at position 34 in tRNA-Arg2C, and Inosine is read as “G” in Sanger sequencing. WT strains were cultured in the presence of an iron chelator 2’,2’-dipyridyl (Dip), and the editing at position 34 in tRNA-Arg2C was measured by Sanger sequencing. The concentration of Dip is indicated on the left. Under all conditions, position 34 is fully edited to inosine (I)
