# Supplementary material for: Sequential action of a tRNA base editor in conversion of cytidine to pseudouridine
Source: Nat Commun. 2022 Oct 11;13:5994. doi: 10.1038/s41467-022-33714-x (PMC9553926; doi:10.1038/s41467-022-33714-x)
Supplement: Supplementary file 3 — Description of Additional Supplementary Files [file 41467_2022_33714_MOESM3_ESM.pdf]

Description of Additional Supplementary Files

Supplementary Data 1 Structural homology search using full-length TrcP as a query

Supplementary Data 2 Structural homology search using the TrcP-CDA domain as a query

Supplementary Data 3 Strain list

Supplementary Data 4 Plasmid list

Supplementary Data 5 Primer list

Supplementary Data 6 Parameters of mass spectrometry for dynamic MRM analyses

Supplementary Data 7 A result of Blast search using the TrcP-NTD as a query

Supplementary Data 8 Phylogenetic distribution of TrcP-NTD homologs

Supplementary Data 9 Structural model of full-length TrcP

Supplementary Data 10 Structural model of TrcP-NTD

Supplementary Data 11 Structural model of TrcP-NTD without the long helical domain
